# Facile Synthesis and Characterization of Novel Analcime/Sodium Magnesium Aluminum Silicon Silicate Nanocomposite for Efficient Removal of Methylene Blue Dye from Aqueous Media

**DOI:** 10.3390/molecules30071488

**Published:** 2025-03-27

**Authors:** Ehab A. Abdelrahman, Zahrah Alqahtani, Mortaga M. Abou-Krisha, Fawaz A. Saad, Reem K. Shah

**Affiliations:** 1 Department of Chemistry, College of Science, Imam Mohammad Ibn Saud Islamic University (IMSIU), Riyadh 11623, Saudi Arabia; 2 Department of Physics, Faculty of Science, Taif University, Taif 21944, Saudi Arabia; 3 Department of Chemistry, Faculty of Science, Umm Al-Qura University, Makkah 21955, Saudi Arabia

**Keywords:** methylene blue dye, adsorption, analcime/sodium magnesium aluminum silicon silicate nanocomposite, kinetics and isotherms

## Abstract

Methylene blue dye, commonly used in various industries, poses significant risks to both human health and the environment due to its persistence, toxicity, and potential to disrupt aquatic ecosystems. Exposure can cause severe health conditions such as methemoglobinemia, while its stability and solubility allow it to persist in natural water systems, reducing oxygen levels and harming aquatic life. In this study, novel analcime/sodium magnesium aluminum silicon silicate nanocomposites (Z1 and Z2) were synthesized via a controlled hydrothermal method, where Z1 and Z2 were synthesized in the absence and presence of polyethylene glycol as a template, respectively. X-ray diffraction (XRD) analysis confirmed the formation of crystalline phases of analcime and sodium magnesium aluminum silicon silicate. The average crystallite size of the Z1 nanocomposite is 75.30 nm, whereas the Z2 nanocomposite exhibits a smaller average crystallite size of 60.27 nm due to the template effect. Field emission scanning electron microscopy (FE-SEM) revealed that Z2 exhibited more uniform and well-dispersed particles compared to Z1. Energy-dispersive X-ray spectroscopy (EDX) confirmed the elemental composition, showing higher sodium content and optimized incorporation of aluminum and silicon in Z2. High-resolution transmission electron microscopy (HR-TEM) demonstrated that Z2 had well-defined spherical particles, indicating improved structural control. The maximum adsorption capacities were 230.95 mg/g for Z1 and 290.69 mg/g for Z2. The adsorption process was exothermic, spontaneous, and chemical in nature, following the pseudo-second-order kinetic model and Langmuir isotherm, confirming monolayer adsorption on homogeneous surfaces.

## 1. Introduction

Water contamination by organic dyes is a widespread environmental problem. It is primarily driven by the discharge of untreated or inadequately treated wastewater from industrial processes [1,2,3]. Major contributors include the textile, paper, leather, and dye manufacturing industries, which use large quantities of synthetic dyes during production. Inadequate wastewater treatment systems and poor regulatory enforcement exacerbate this issue. As a result, colored effluents are allowed to enter water bodies. Agricultural runoff containing dye-based pesticides contributes to dye pollution in aquatic ecosystems. Improper disposal of dye-containing household products, such as detergents and cosmetics, also adds to this contamination [4,5]. The presence of organic dyes in water bodies poses serious risks to the environment and human health. Environmentally, dyes hinder sunlight penetration, disrupting photosynthesis and reducing oxygen levels, which affects aquatic life. Many dyes are chemically stable and resistant to natural degradation, prolonging their environmental persistence. Moreover, certain dyes and their breakdown products are toxic to aquatic organisms, impairing their growth, reproduction, and survival [6,7]. For humans, exposure to organic dyes through contaminated water is linked to severe health issues. This is because many dyes are classified as carcinogenic, mutagenic, or teratogenic. Prolonged exposure can cause skin and eye irritation, as well as respiratory problems. It may also lead to long-term conditions such as liver and kidney damage due to the accumulation of toxic metabolites [8,9,10]. These dangers emphasize the importance of effective remediation strategies to remove dyes from water sources. Methylene blue dye is commonly used in various industries, including textiles, medicine, and biological research. However, its uncontrolled release into the environment presents significant risks. In aquatic ecosystems, methylene blue reduces dissolved oxygen levels, affecting aquatic organisms and disrupting ecological balance. Its high solubility and chemical stability allow it to persist in water, making it challenging to degrade naturally [11,12]. In humans, methylene blue exposure through contaminated water can cause adverse health effects. Acute exposure may lead to symptoms such as nausea, vomiting, increased heart rate, and confusion. Prolonged exposure is associated with methemoglobinemia, a condition that reduces the oxygen-carrying capacity of blood [13,14]. These environmental and human risks highlight the need for effective removal methods. Several techniques have been developed for the removal of organic dyes from water, each offering different levels of efficiency and feasibility. Adsorption is a widely used method where dye molecules adhere to the surface of an adsorbent [15]. Photocatalytic degradation is an advanced oxidation process that utilizes light-activated catalysts to break down organic dyes in wastewater, but its disadvantages include high energy requirements, limited efficiency under natural sunlight, potential formation of toxic byproducts, and the challenge of recovering or reusing the catalyst [16]. Techniques like reverse osmosis and nanofiltration are effective, but expensive and prone to fouling [17,18]. Electrochemical methods degrade dye molecules using electric current, but require high energy input [19,20]. Bioremediation uses microorganisms to degrade dyes biologically, though it is often limited by environmental conditions and slow degradation rates [21,22]. Adsorption is considered superior to other dye removal methods due to its simplicity, low cost, and effectiveness in treating large volumes of wastewater. Unlike chemical precipitation, it does not produce large amounts of harmful sludge. It is more energy-efficient than electrochemical methods and does not require expensive equipment, as seen in membrane filtration. Adsorption is also versatile, as it can remove a wide range of dyes at low concentrations. Many adsorbents, including natural and synthetic materials, can be regenerated and reused, enhancing the sustainability of the process [23,24]. Metal oxide nanoparticles are highly effective adsorbents due to their large surface area, high reactivity, and tunable surface properties. Their nanoscale dimensions provide abundant active sites for dye adsorption. Common metal oxides such as ZnO, TiO_2_, and Fe_3_O_4_ exhibit strong adsorption capabilities and can be easily modified to improve performance. Their chemical stability and resistance to degradation make them ideal for long-term applications in water treatment [25,26,27]. Zeolites and sodium metal silicates, known for their well-defined microporous structure and high ion-exchange capacity, are effective adsorbents for dye removal. Their negatively charged framework enables the adsorption of positively charged dye molecules through electrostatic interactions and ion exchange [28]. Zeolite-based composites, when combined with materials such as chitosan and metal oxides, exhibit enhanced adsorption efficiency due to additional functional groups and active sites. This synergy allows for the effective removal of both cationic and anionic dyes, making zeolite composites a promising option for water purification [29,30]. This research develops a novel analcime/sodium magnesium aluminum silicon silicate nanocomposite through a controlled hydrothermal method. The incorporation of sodium, magnesium, aluminum, and silicon precursors forms a crystalline material with enhanced adsorption properties. Polyethylene glycol, used as a structure-directing agent, optimizes the nanocomposite’s morphology and stability, increasing active sites for dye adsorption. The innovation lies in both the synthesis of this nanocomposite and its application for the efficient removal of methylene blue dye from aqueous media, with its unique structure enhancing adsorption through ion exchange.

## 2. Results and Discussion

### 2.1. Characterization

**Figure 1:**
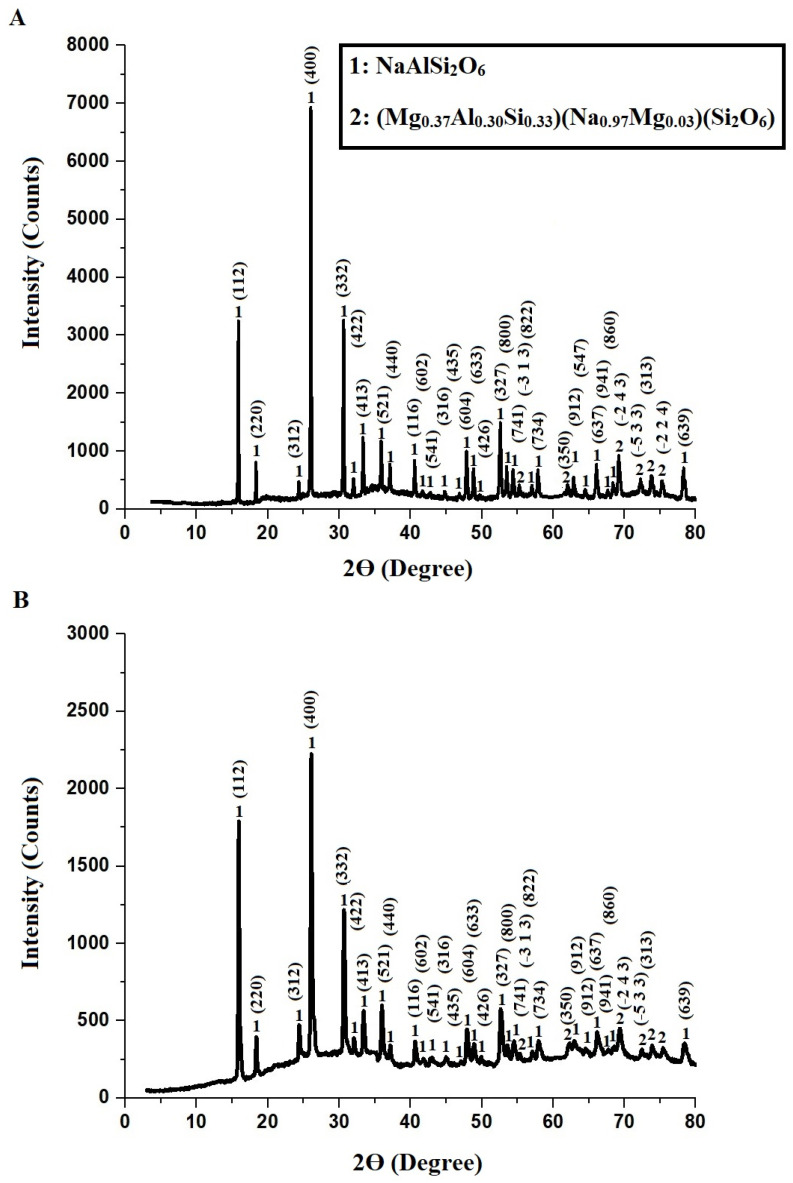
XRD of Z1 (**A**) and Z2 (**B**) nanocomposites.

Figure 1 illustrates the XRD patterns of the Z1 (Figure 1A) and Z2 (Figure 1B) nanocomposites, where Z1 denotes the nanocomposite synthesized without a template, and Z2 refers to the nanocomposite synthesized using polyethylene glycol 400 as a template. The XRD profiles reveal distinct diffraction peaks corresponding to the crystalline phases of analcime (NaAlSi_2_O_6_, tetragonal system, JCPDS No. 01-073-6448) and sodium magnesium aluminum silicon silicate ((Mg_0.37_Al_0.30_Si_0.33_)(Na_0.97_Mg_0.03_)(Si_2_O_6_), monoclinic system, JCPDS No. 01-077-9820). The characteristic diffraction angles (2θ) associated with analcime are observed at 15.83°, 18.26°, 24.33°, 26.01°, 30.64°, 31.99°, 33.36°, 35.88°, 37.14°, 40.60°, 41.55°, 42.69°, 44.79°, 46.80°, 47.84°, 48.90°, 49.63°, 52.57°, 53.52°, 54.35°, 56.98°, 57.91°, 62.85°, 64.42°, 65.99°, 67.67°, 68.42°, and 78.27°, corresponding to the Miller indices (112), (220), (312), (400), (332), (422), (413), (521), (440), (116), (602), (541), (316), (435), (604), (633), (426), (327), (800), (741), (822), (734), (912), (547), (637), (941), (860), and (639), respectively. For sodium magnesium aluminum silicon silicate, the diffraction angles (2θ) are 55.30°, 62.11°, 69.26°, 72.30°, 73.87°, and 75.33°, with the corresponding Miller indices being (−3 1 3), (350), (−2 4 3), (−5 3 3), (313), and (−2 2 4), respectively. Both Z1 and Z2 nanocomposites exhibit the same crystalline phases corresponding to analcime and sodium magnesium aluminum silicon silicate, as confirmed by the characteristic diffraction peaks in the XRD patterns. However, a notable difference is observed in the crystallite size. Z1 shows an average crystallite size of 75.30 nm, while Z2 displays a smaller crystallite size of 60.27 nm. This reduction is attributed to the use of polyethylene glycol 400 as a structure-directing agent in the synthesis of Z2, which limits crystal growth and improves particle dispersion.

**Figure 2:**
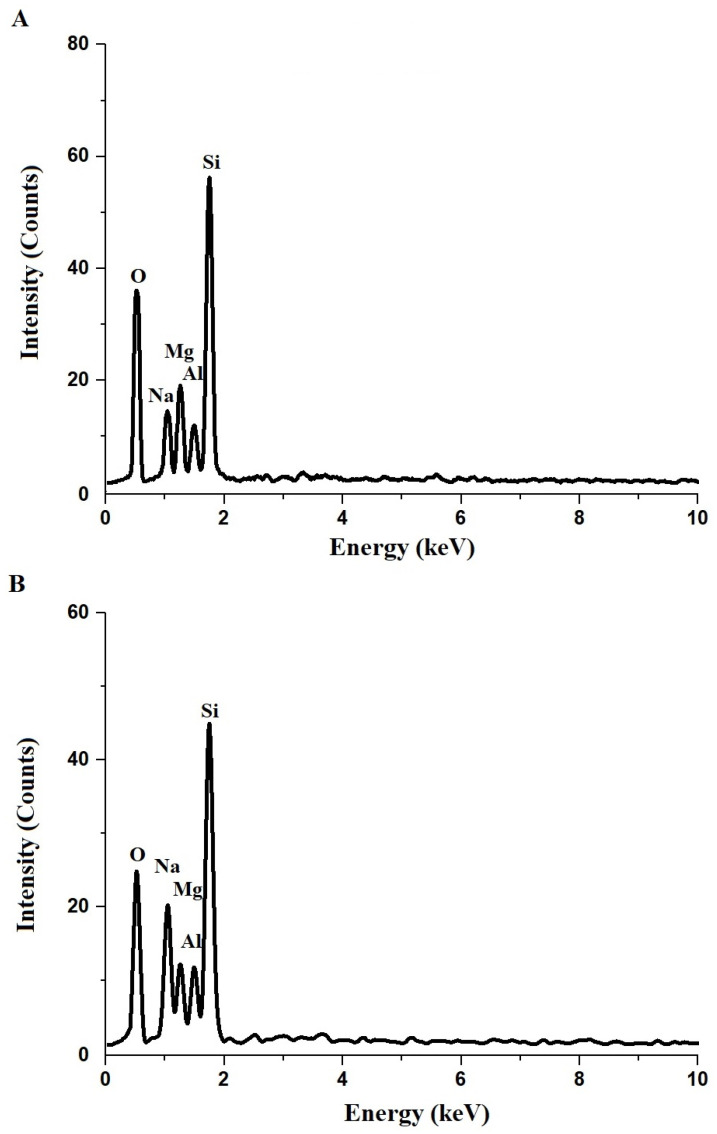
EDX patterns of Z1 (**A**) and Z2 (**B**) nanocomposites.

Figure 2 shows the EDX patterns of the Z1 (Figure 2A) and Z2 (Figure 2B) nanocomposites, providing insight into the elemental composition of the samples. The peaks corresponding to oxygen (O), sodium (Na), magnesium (Mg), aluminum (Al), and silicon (Si) are clearly visible in both patterns, confirming their presence in the nanocomposites. Table 1 summarizes the atomic percentages of the key elements in Z1 and Z2. The atomic percentage of oxygen in Z1 is 59.3%, while in Z2, it is 55.2%. Sodium shows a significant increase from 8.1% in Z1 to 11.5% in Z2, while magnesium decreases from 8.6% to 6.0%. Aluminum content increases from 3.4% in Z1 to 4.9% in Z2, and silicon shows a slight increase from 20.6% to 22.4%. The changes in the elemental composition can be attributed to the role of polyethylene glycol 400, which acts as a template during the synthesis of Z2, influencing the distribution and incorporation of elements. The decrease in oxygen content in Z2 compared to Z1 suggests a modification in the structural environment due to the presence of the template, potentially altering the surface oxygen species. The significant increase in sodium content indicates enhanced incorporation of sodium ions, possibly due to improved ion diffusion and stabilization facilitated by the template. The decrease in magnesium content in Z2 may result from a redistribution of cations within the framework, while the increased aluminum and silicon contents reflect enhanced crystallization and condensation of the silicate and aluminosilicate phases. Overall, the presence of polyethylene glycol 400 modifies the synthesis dynamics, leading to altered elemental distributions and potentially improved structural and adsorption properties.

**Figure 3:**
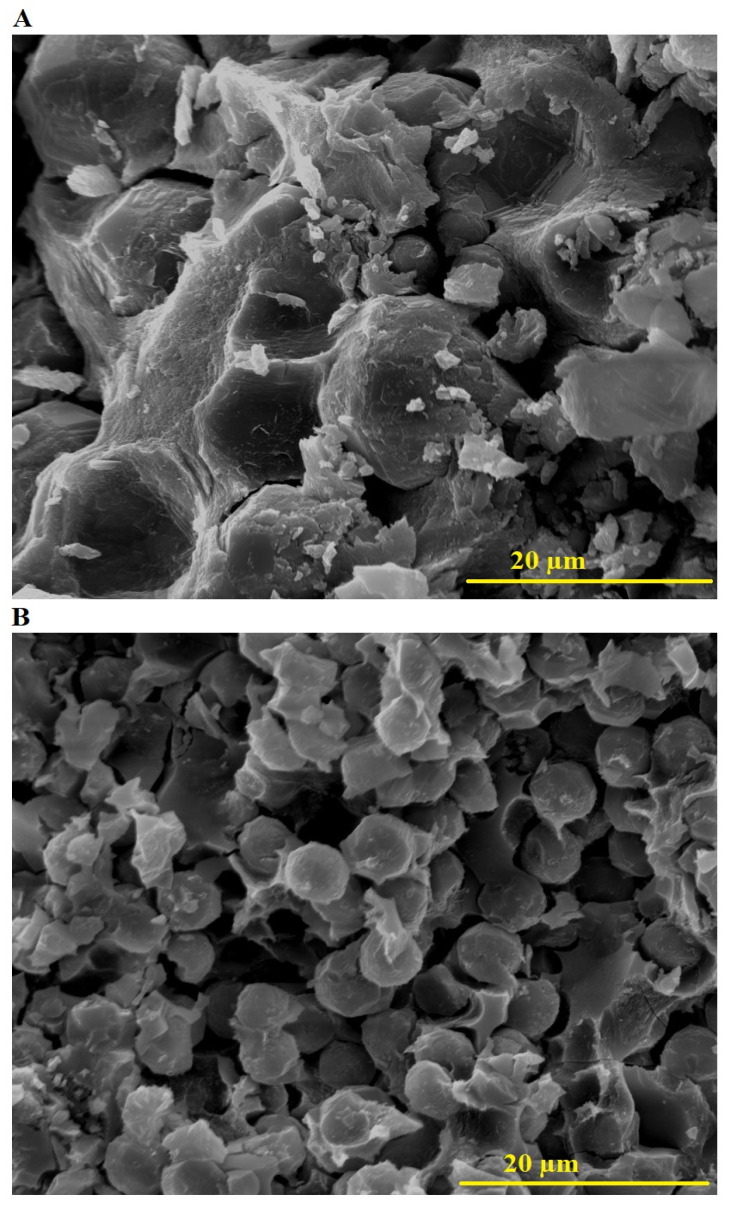
FE-SEM images of Z1 (**A**) and Z2 (**B**) nanocomposites.

Figure 3 displays the FE-SEM images of the Z1 (Figure 3A) and Z2 (Figure 3B) nanocomposites, highlighting notable differences in their morphologies. The Z1 nanocomposite shows a rough and compact structure dominated by large, irregular, and tightly packed plate-like formations, suggesting uncontrolled particle growth and significant agglomeration during synthesis. In contrast, the Z2 nanocomposite synthesized using polyethylene glycol 400 exhibits a more organized and well-dispersed morphology characterized by smaller, spherical, and granular particles. This morphological transformation can be attributed to the role of polyethylene glycol 400 as a structure-directing agent, which promotes better control over crystal nucleation and growth while minimizing agglomeration. As a result, the use of polyethylene glycol 400 leads to a more refined and uniform structure in Z2 compared to the coarser and less controlled morphology observed in Z1.

**Figure 4:**
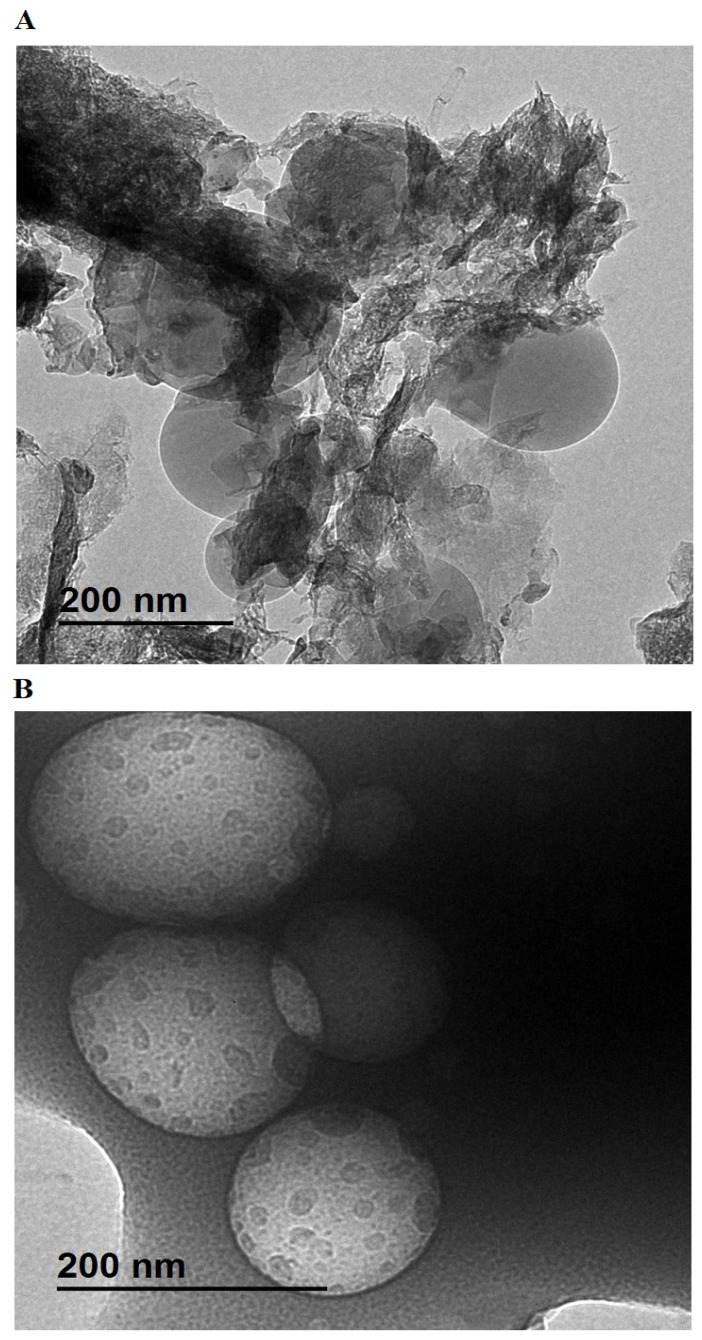
HR-TEM images of Z1 (**A**) and Z2 (**B**) nanocomposites.

Figure 4 presents HR-TEM images of the Z1 (Figure 4A) and Z2 (Figure 4B) nanocomposites, revealing distinct morphological differences influenced by the synthesis conditions. In the Z1 sample, large and irregular sheet-like structures are observed, suggesting agglomeration and poor control over particle formation. These sheets appear to consist of overlapping and stacked layers with varying thicknesses, indicating a less defined and more disordered structure. In contrast, the Z2 sample exhibits spherical particles with well-defined boundaries and relatively uniform sizes, reflecting improved structural control. The spherical shape in Z2 is attributed to the templating effect of polyethylene glycol 400, which directs the nucleation and growth of particles, preventing excessive agglomeration and promoting the formation of discrete and homogeneous structures. The morphological transition from large, irregular sheets in Z1 to well-dispersed spherical particles in Z2 demonstrates the significant role of the template in refining the growth mechanism and achieving better-defined nanostructures.

### 2.2. Removal of Methylene Blue Dye from Aqueous Media

The removal percentage of methylene blue dye (% R) and the adsorption capacity of the adsorbent (Q) were calculated using Equation (1) and Equation (2), respectively [31].

(1) $$\%\;R=\frac{C_{o}-C_{e}}{C_{o}}\times 100\;$$

(2) $$Q=\left(C_{o}-C_{e}\right)\times \frac{V}{W}$$

In these equations, C_o_ is the initial dye concentration (mg/L), V is the volume of the solution (L), W is the mass of the adsorbent (g), and C_e_ is the equilibrium dye concentration (mg/L).

#### 2.2.1. Effect of pH

**Figure 5:**
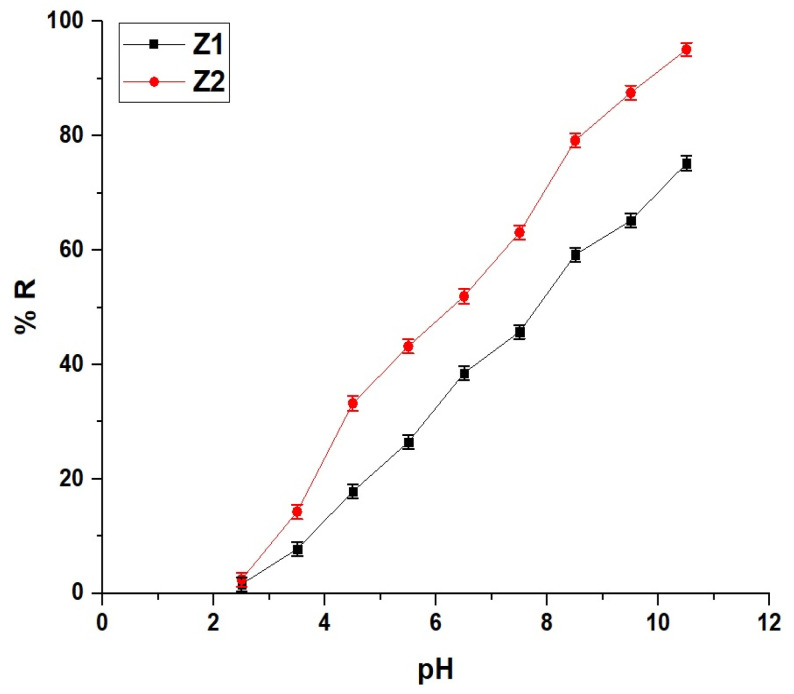
Effect of pH on the removal efficiency (% R) of methylene blue dye using Z1 and Z2 nanocomposites.

**Figure 6:**
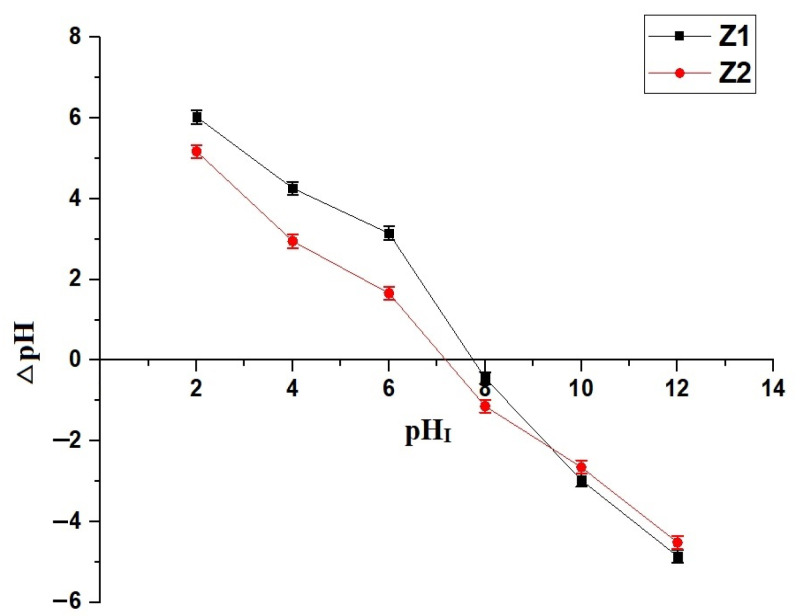
Determination of the point of zero charge (pH_PZC_) based on ΔpH variation as a function of initial pH (pH_I_) for Z1 and Z2 nanocomposites.

Figure 5 and Figure 6 demonstrate the influence of pH on the adsorption performance of Z1 and Z2 nanocomposites for methylene blue dye removal and the determination of their points of zero charge (pH_PZC_), respectively. The removal efficiency (% R) of methylene blue dye, as shown in Figure 5, increases steadily with rising pH for both Z1 and Z2, with Z2 consistently achieving higher removal percentages across the pH range. At pH 2.5, Z1 exhibits a removal efficiency of 1.64%, while Z2 reaches 2.45%, indicating a more effective adsorption efficiency in the early acidic region. This trend continues at higher pH values, with Z2 achieving 33.25% dye removal at pH 4.5 compared to 17.83% for Z1. As the pH reaches 7.5, Z1 shows 45.75% removal, whereas Z2 significantly outperforms it with 63.12% removal. This gap widens further in alkaline conditions, with Z2 achieving 95.09% removal at pH 10.5 compared to 75.22% for Z1, reflecting the enhanced adsorption properties of Z2. The pH_PZC_ values, as illustrated in Figure 6, are 7.70 for Z1 and 7.16 for Z2, marking the pH at which the surface charge of the nanocomposites becomes neutral. Below their respective pH_PZC_ values, the surfaces of both nanocomposites are positively charged, leading to reduced electrostatic attraction with the cationic methylene blue dye. As the pH surpasses the pH_PZC_, the surface charge becomes increasingly negative, enhancing dye adsorption due to stronger electrostatic interactions. Z2’s lower pH_PZC_ value implies an earlier onset of negative surface charge, correlating with its superior performance in dye removal across the tested pH range. The combination of improved surface charge behavior and morphological advantages explains the higher adsorption capacity of Z2 compared to Z1.

#### 2.2.2. Effect of Contact Time

**Figure 7:**
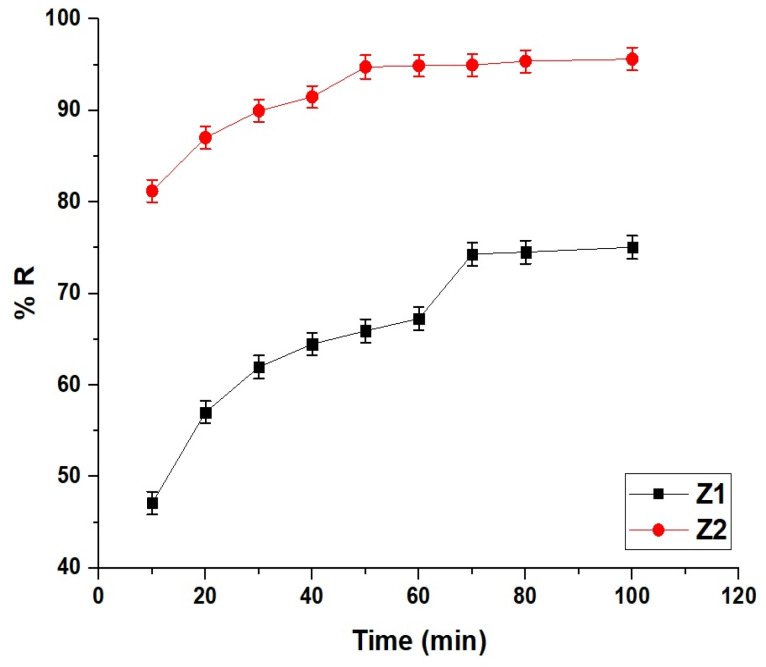
Comparative removal efficiency (% R) of Z1 and Z2 nanocomposites over time.

Figure 7 illustrates the comparative removal efficiency of methylene blue dye by the Z1 and Z2 nanocomposites over time. At 10 min, the Z1 nanocomposite achieved a removal efficiency of 47.12%, while Z2 exhibited a significantly higher efficiency of 81.20%, demonstrating the superior initial adsorption efficiency of Z2. At 50 min, Z1 reached a removal efficiency of 65.93%, whereas Z2 achieved 94.75%, indicating that Z2 reached its equilibrium at 50 min while continuing to display higher removal efficiency compared to Z1. At 70 min, Z1 reached its equilibrium with a removal efficiency of 74.31%. Beyond these equilibrium times, the removal efficiency did not exhibit significant changes due to the saturation of the adsorbents. The overall comparison highlights that Z2 not only adsorbs the dye at a faster rate, but also achieves a higher final removal efficiency compared to Z1. The difference can be attributed to the enhanced structural and adsorption characteristics of Z2, allowing it to outperform Z1 in both kinetics and efficiency within the same experimental conditions.

**Figure 8:**
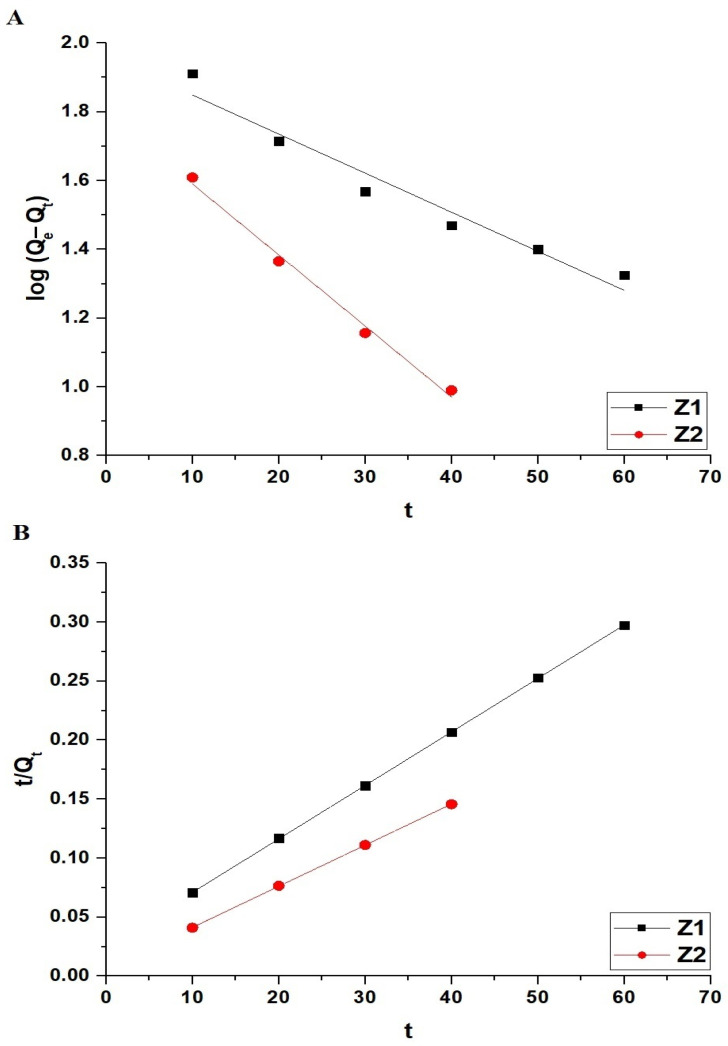
Kinetic modeling of methylene blue dye adsorption onto Z1 and Z2 nanocomposites. (**A**) Pseudo-first-order kinetic model and (**B**) pseudo-second-order kinetic model fitting curves.

The adsorption kinetics of methylene blue dye onto Z1 and Z2 nanocomposites were evaluated using the pseudo-first-order and pseudo-second-order models, as shown in Figure 8A,B, respectively. The results are summarized in Table 2. The pseudo-first-order model is described by Equation (3) [32].

(3) $$\log\left(Q_{e}-Q_{t}\right)={\mathrm{logQ}}_{e}-\frac{K_{1}}{2.303}t\;$$

The pseudo-second-order model is described by Equation (4) [32].

(4) $$\frac{t}{Q_{t}}=\frac{1}{K_{2}Q_{e}^{2}}+\frac{1}{Q_{e}}t\;$$

In these equations, Q_e_ (mg/g) is the equilibrium adsorption capacity, Q_t_ (mg/g) is the adsorption capacity at time t (min), K_1_ (1/min) is the rate constant for the pseudo-first-order model, and K_2_ (g/mg·min) is the rate constant for the pseudo-second-order model.

As depicted in Figure 8, the kinetic data for both Z1 and Z2 nanocomposites were analyzed, revealing better agreement with the pseudo-second-order model based on the experimental adsorption capacity values (Q_Exp_) and the high correlation coefficients (R^2^ = 0.9999) in Table 2. The values of Q_e_ obtained from the pseudo-second-order model closely match the experimental values, confirming that the adsorption process predominantly follows the pseudo-second-order kinetics, suggesting chemisorption as the rate-limiting mechanism.

#### 2.2.3. Effect of Temperature

**Figure 9:**
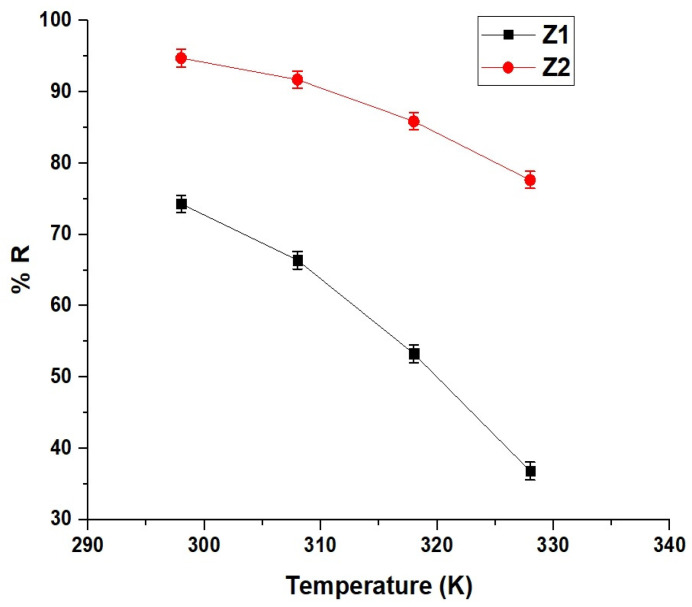
Effect of temperature on the removal efficiency (% R) of Z1 and Z2 nanocomposites for methylene blue dye.

Figure 9 demonstrates the effect of temperature on the removal efficiency of methylene blue dye by Z1 and Z2 nanocomposites. At 298 K, Z1 exhibits a removal efficiency of 74.31%, while Z2 achieves a significantly higher efficiency of 94.75%, highlighting the superior performance of Z2 at lower temperatures. As the temperature increases to 328 K, the removal efficiency decreases substantially for both samples, with Z1 dropping to 36.83% and Z2 to 77.64%. The greater decline in Z1 compared to Z2 indicates that Z1 is more sensitive to temperature increases. This behavior can be attributed to the exothermic nature of the adsorption process, where higher temperatures hinder dye adsorption by reducing the interaction between the adsorbent and the dye molecules.

The thermodynamic parameters for the adsorption of methylene blue dye onto Z1 and Z2 nanocomposites were determined using Equations (5)–(7) [33].

(5) $${\mathrm{lnK}}_{d}=\frac{△S^{o}}{R}-\frac{△H^{o}}{\mathrm{RT}}\;$$

(6) $$△G^{o}=△H^{o}-T△S^{o}\;$$

(7) $$K_{d}=\frac{Q_{e}}{C_{e}}$$

In these equations, K_d_ is the distribution coefficient, ΔS^o^ is the standard entropy change, ΔH^o^ is the standard enthalpy change, R is the universal gas constant, ΔG^o^ is the standard Gibbs free energy change, and T is the temperature in Kelvin.

**Figure 10:**
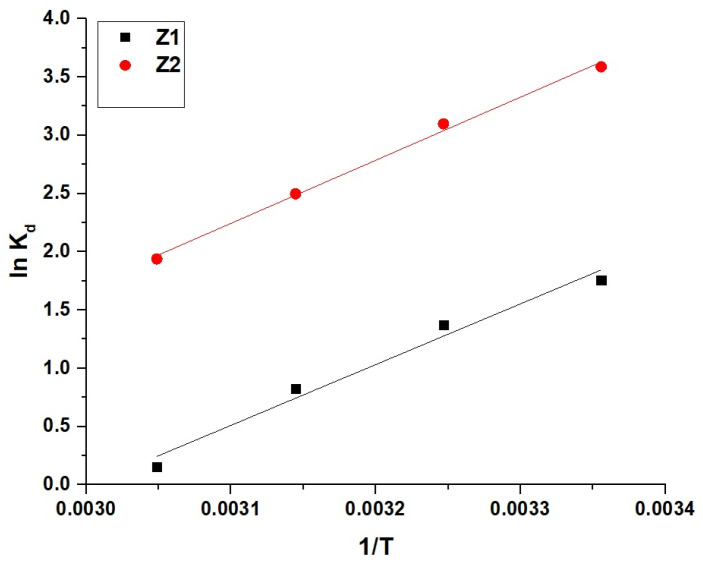
Van’t Hoff plots for the adsorption of methylene blue dye onto Z1 and Z2 nanocomposites.

As shown in Figure 10, the Van’t Hoff plots reveal a linear relationship between lnK_d_ and 1/T, confirming the temperature dependence of the adsorption process. The calculated thermodynamic parameters presented in Table 3 indicate that the adsorption is chemical in nature, as the values of ΔH^o^ for both Z1 and Z2 exceed 40 kJ/mol, with −43.57 kJ/mol for Z1 and −45.25 kJ/mol for Z2. The negative values of ΔH^o^ demonstrate that the adsorption process is exothermic. Furthermore, the negative values of ΔG^o^ at all temperatures confirm that the adsorption is spontaneous, with Z2 showing slightly less negative values than Z1, indicating its relatively higher stability across the temperature range. The positive values of ΔS^o^ for both nanocomposites signify increased randomness at the solid–liquid interface, making the adsorption process thermodynamically feasible. Overall, the combination of chemical, exothermic, spontaneous, and feasible characteristics highlights the efficient interaction between methylene blue dye and both nanocomposites under the given conditions.

#### 2.2.4. Effect of Concentration

**Figure 11:**
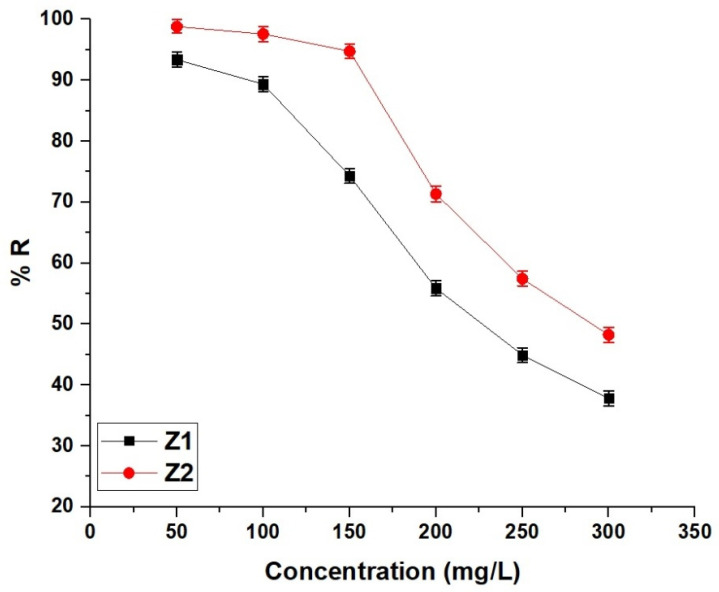
Effect of initial methylene blue dye concentration on the removal efficiency of Z1 and Z2 nanocomposites.

Figure 11 illustrates the effect of initial methylene blue dye concentration on the removal efficiency of Z1 and Z2 nanocomposites. At an initial concentration of 50 mg/L, Z1 achieves a removal efficiency of 93.36%, while Z2 demonstrates superior performance with 98.80%. As the concentration increases to 100 mg/L, the removal efficiency slightly decreases to 89.36% for Z1 and 97.57% for Z2. This decreasing trend continues more significantly at 150 mg/L, where Z1 and Z2 exhibit removal efficiencies of 74.31% and 94.75%, respectively. At 200 mg/L, the efficiencies further decline, with Z1 reaching 55.88% and Z2 maintaining 71.34%. At 250 mg/L, Z1 records 44.93% and Z2 achieves 57.45%, and at the highest tested concentration of 300 mg/L, the removal efficiencies drop to 37.82% for Z1 and 48.23% for Z2. The observed decline in removal efficiency with increasing concentration is attributed to the saturation of available adsorption sites, limiting the ability of the nanocomposites to accommodate additional dye molecules. Throughout the concentration range, Z2 consistently outperforms Z1, highlighting its enhanced adsorption capacity and greater resistance to efficiency loss under higher dye concentrations.

**Figure 12:**
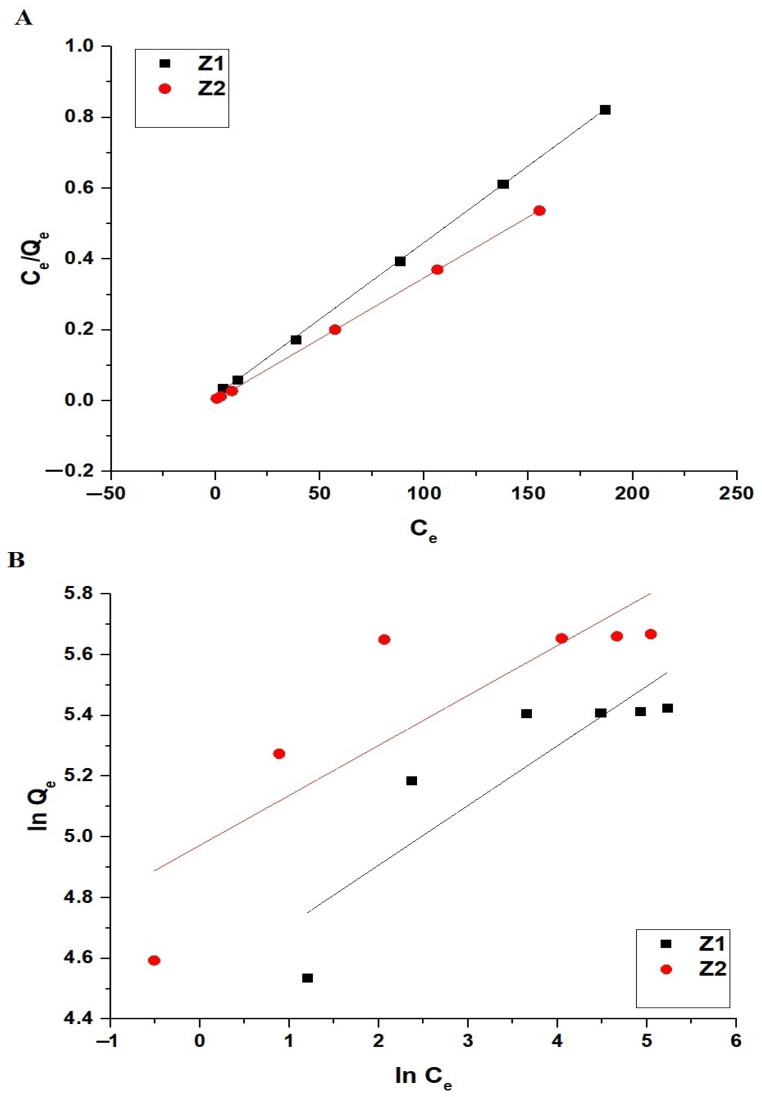
Linear fitting of adsorption isotherms for methylene blue dye onto Z1 and Z2 nanocomposites: (**A**) Langmuir isotherm and (**B**) Freundlich isotherm.

The adsorption data were analyzed using the Langmuir (Equation (8)) and Freundlich (Equations (9) and (10)) isotherms [32]. Also, Figure 12A,B represents the Langmuir and Freundlich isotherms, respectively.

(8) $$\frac{C_{e}}{Q_{e}}=\frac{1}{K_{3}Q_{\max}}+\frac{C_{e}}{Q_{\max}}$$

(9) $${\mathrm{lnQ}}_{e}={\mathrm{lnK}}_{4}+\frac{1}{n}{\mathrm{lnC}}_{e}\;$$

(10) $$Q_{\max}=K_{4}\left(C_{o}^{1/n}\right)\;$$

In these equations, C_e_ is the equilibrium concentration of the dye in solution, Q_e_ is the amount of dye adsorbed at equilibrium, K_3_ is the Langmuir constant related to adsorption affinity, Q_max_ is the maximum adsorption capacity, K_4_ is the Freundlich constant related to adsorption capacity, and 1/n is a dimensionless parameter indicating adsorption intensity.

As shown in Figure 12, the adsorption data fit both the Langmuir and Freundlich isotherms, but the high values of R^2^ for the Langmuir model, particularly 0.9997 for Z1 and 0.9999 for Z2, as presented in Table 4, confirm that the adsorption follows the Langmuir isotherm. This indicates that the adsorption occurs on a homogeneous surface with monolayer coverage, suggesting uniform adsorption sites without significant interactions between adsorbed molecules. The maximum adsorption capacities derived from the Langmuir model are 230.95 mg/g for Z1 and 290.69 mg/g for Z2, highlighting the superior adsorption capacity of Z2 compared to Z1, which can be attributed to its enhanced structural properties and surface interaction with the dye molecules.

Table 5 presents the maximum adsorption capacities (Q_max_) of various adsorbents for the removal of methylene blue dye [34,35,36,37,38,39,40]. The adsorption capacities range from 24.39 mg/g for NaX zeolite to 290.69 mg/g for the Z2 nanocomposite developed in this study. Among the listed materials, activated coconut shells, NaX zeolite, and graphene oxide/ZnTiO_3_/TiO_2_ composites demonstrate relatively low adsorption capacities of 30.30 mg/g, 24.39 mg/g, and 78.00 mg/g, respectively. Although the activated carbon/sodium lauryl sulfate composite and ZnO/chitosan composite exhibit higher adsorption capacities of 232.5 mg/g and 97.93 mg/g, they are still surpassed by both Z1 and Z2 nanocomposites. Z1 shows a Q_max_ of 230.95 mg/g, while Z2 exhibits the highest capacity at 290.69 mg/g. The superior adsorption performance of Z1 and Z2 can be attributed to their unique structural features and enhanced interactions with methylene blue dye. The synthesis process incorporating polyethylene glycol 400 in Z2 plays a critical role in optimizing the nanocomposite’s surface characteristics, including higher adsorption sites and improved dispersion, which facilitate better dye uptake compared to other adsorbents. Additionally, the higher crystallinity and surface functionality of Z1 and Z2 contribute to stronger chemical interactions between the dye molecules and the adsorbent surface. As a result, Z1 and Z2 outperform conventional materials like zeolites, composites, and activated carbons in terms of adsorption efficiency, making them highly effective for methylene blue removal.

The BET surface area of the Z1 nanocomposite was found to be 84.73 m^2^/g, while Z2 exhibited a higher surface area of 123.16 m^2^/g, confirming that the utilization of polyethylene glycol 400 as a template enhances surface porosity and increases the number of active sites available for adsorption. This increase in surface area directly contributes to the superior adsorption capacity (Q_max_) of Z2 (290.69 mg/g) compared to Z1 (230.95 mg/g), as the larger surface allows for more dye molecules to interact with the adsorbent.

The oxidation states of the elements in both Z1 and Z2 nanocomposites are expected to be Na^+^, Mg^2+^, Al^3+^, and Si^4+^, which correspond to their common stable valence states in analcime and sodium magnesium aluminum silicon silicate phases. Since both samples exhibit the same phases, the oxidation states are not the key factor influencing the difference in methylene blue removal. Instead, the enhanced removal performance of Z2 is mainly attributed to its higher surface area, smaller crystallite size, better dispersion, and greater sodium content, all of which improve adsorption efficiency.

## 3. Experimental

### 3.1. Materials

The chemicals used in this study were obtained from Sigma-Aldrich (St. Louis, MO, USA). Aluminum nitrate nonahydrate (Al(NO_3_)_3_·9H_2_O), sodium metasilicate pentahydrate (Na_2_SiO_3_·5H_2_O), magnesium nitrate hexahydrate (Mg(NO_3_)_2_·6H_2_O), sodium hydroxide (NaOH), polyethylene glycol 400 (H(OCH_2_CH_2_)_n_OH), and hydrochloric acid (HCl) were employed as key reagents for nanocomposite synthesis and treatment processes. Potassium chloride (KCl) was used as an electrolyte for the determination of point of zero charge (pH_PZC_). Methylene blue dye (C_16_H_18_ClN_3_S) was used as the model contaminant in adsorption experiments. All chemicals were of analytical grade with purity ≥98% and were used as received without further purification.

### 3.2. Synthesis of Analcime/Sodium Magnesium Aluminum Silicon Silicate Nanocomposites

**Figure 13:**
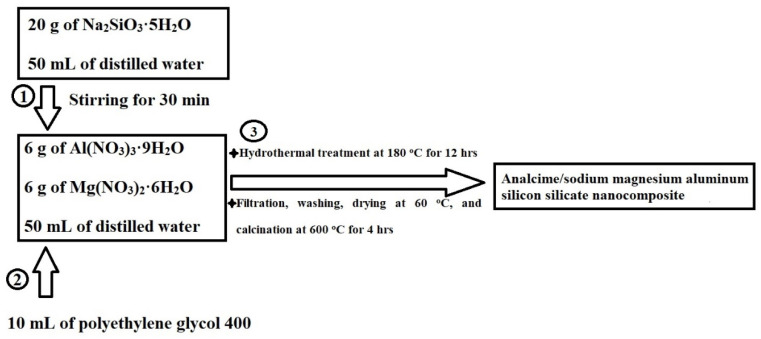
Synthesis process of analcime/sodium magnesium aluminum silicon silicate nanocomposite using polyethylene glycol 400.

A solution was prepared by dissolving 20 g of Na_2_SiO_3_·5H_2_O in 50 mL of distilled water. Separately, 6 g of Al(NO_3_)_3_·9H_2_O and 6 g of Mg(NO_3_)_2_·6H_2_O were dissolved in 50 mL of distilled water. The two solutions were combined under continuous stirring for 30 min. To the resulting mixture, 10 mL of polyethylene glycol 400 was added, and the stirring continued for an additional 30 min. The mixture was subjected to hydrothermal treatment at 180 °C for 12 h using a 170 mL Teflon-lined stainless-steel autoclave. The formed precipitate was separated by filtration, washed thoroughly with distilled water, dried at 60 °C, and calcined at 600 °C for 4 h to yield the analcime/sodium magnesium aluminum silicon silicate nanocomposite (abbreviated as Z2). The calcination temperature of 600 °C was chosen to ensure the complete removal of the organic component (polyethylene glycol 400), as well as to promote the formation of a stable crystalline structure necessary for enhanced adsorption performance. The same procedure was followed without calcination, but polyethylene glycol 400 was replaced with 10 mL of distilled water to yield the analcime/sodium magnesium aluminum silicon silicate nanocomposite (abbreviated as Z1). Figure 13 illustrates the synthesis process of the analcime/sodium magnesium aluminum silicon silicate nanocomposite using polyethylene glycol 400.

### 3.3. Instrumentation

The crystal structural properties of all synthesized samples were analyzed using an X-ray diffraction diffractometer (XRD, X′Pert PRO, PANalytical, Almelo, The Netherlands). The surface morphology and elemental composition of the samples were examined using a field emission scanning electron microscope (FE-SEM) equipped with energy-dispersive X-ray spectroscopy (EDX) (Quanta 250 FEG, Thermo Fisher Scientific, Waltham, MA, USA). High-resolution transmission electron microscopy (HR-TEM, JEM-2100Plus, JEOL Ltd., Tokyo, Japan) was employed to investigate the morphology of the synthesized samples. The concentration of methylene blue dye in the adsorption studies was determined using a UV-Vis spectrophotometer (Cintra 3030, GBC, Melbourne, Australia).

### 3.4. Removal of Methylene Blue Dye from Aqueous Media

The removal of methylene blue dye from aqueous solutions was investigated under varying experimental conditions, as summarized in Table 6. To study the effect of pH, 0.1 L methylene blue solutions with initial concentrations of 150 mg/L were prepared, and 50 mg of adsorbent was added to each solution at 298 K. The pH was adjusted within the range of 2.5 to 10.5 using hydrochloric acid or sodium hydroxide solutions, and the solutions were stirred using a magnetic stirrer for 360 min. For the effect of contact time, 0.1 L methylene blue solutions with initial concentrations of 150 mg/L were prepared, and 50 mg of adsorbent was added to each solution and stirred at 298 K and pH 10.5 for time intervals ranging from 10 to 100 min. To evaluate the effect of temperature, 0.1 L methylene blue solutions with initial concentrations of 150 mg/L were prepared, and 50 mg of adsorbent was added to each solution. The solutions were stirred at different temperatures ranging from 298 K to 328 K at pH 10.5, with stirring times of 70 min for Z1 and 50 min for Z2. For the effect of the initial methylene blue dye concentration, 0.1 L methylene blue solutions with concentrations varying from 50 mg/L to 300 mg/L were prepared, and 50 mg of adsorbent was added to each solution. The solutions were stirred at 298 K and pH 10.5, with a stirring time of 70 min for Z1 and 50 min for Z2. After adsorption, the adsorbent was separated using a centrifuge, and the concentration of methylene blue dye in the filtrate was measured using a UV-Vis spectrophotometer at 660 nm.

### 3.5. Point of Zero Charge (pH_PZC_) of Nanocomposites

The point of zero charge (pH_PZC_) of the synthesized nanocomposites was determined using the batch adsorption method with KCl as the supporting electrolyte [33]. A series of 50 mL KCl solutions with an initial concentration of 0.01 M were prepared, and their initial pH (pH_I_) was adjusted within the range of 2 to 12 using 0.1 M HCl or NaOH solutions. To each solution, 0.05 g of the nanocomposite was added, and the mixtures were stirred at room temperature for 24 h to allow equilibrium to be reached. The final pH of the solutions (pH_F_) was measured using a calibrated pH meter. The difference between the initial and final pH values (∆pH) was plotted against the initial pH (pH_I_). The point where the ΔpH equals zero corresponds to the point of zero charge (pH_PZC_) of the nanocomposite, indicating the pH at which the surface of the adsorbent has a neutral net charge.

## 4. Conclusions

This study successfully synthesized and characterized novel analcime/sodium magnesium aluminum silicon silicate nanocomposites (Z1 and Z2) using a controlled hydrothermal method, with and without polyethylene glycol 400 as a template. Structural analyses confirmed that both nanocomposites exhibit the same crystalline phases; however, Z2 showed superior properties due to the templating effect. Specifically, Z2 exhibited a smaller average crystallite size (60.27 nm) compared to Z1 (75.30 nm), as confirmed by XRD analysis. The surface morphology analysis via FE-SEM and HR-TEM revealed that Z2 had more uniform, spherical, and well-dispersed particles, while Z1 exhibited aggregated and irregular sheet-like structures. The EDX results demonstrated higher sodium and silicon content in Z2, suggesting enhanced framework incorporation. The BET analysis revealed a significantly higher surface area for Z2 (123.16 m^2^/g) than Z1 (84.73 m^2^/g), providing more active sites for adsorption. These structural and surface enhancements translated into superior adsorption performance, with Z2 achieving a maximum adsorption capacity of 290.69 mg/g, compared to 230.95 mg/g for Z1 toward methylene blue dye. The adsorption process followed the pseudo-second-order kinetic model (R^2^ = 0.9999) and Langmuir isotherm, indicating that chemisorption on a homogeneous monolayer surface was the dominant mechanism. Thermodynamic studies revealed that the adsorption was spontaneous, exothermic, and feasible, with an increase in entropy at the solid–liquid interface. Overall, the enhanced removal efficiency of Z2 can be attributed to its higher surface area, improved morphology, better dispersion, optimized composition, and lower point of zero charge (pH_PZC_ = 7.16 vs. 7.70 for Z1), which promoted stronger electrostatic interactions with cationic methylene blue dye at higher pH values. These results confirm that the use of polyethylene glycol 400 as a template significantly enhances the structural and functional properties of the nanocomposite, making Z2 a promising and efficient adsorbent for methylene blue dye removal from aqueous media.

## Figures and Tables

**Table 1:** Atomic percentages of elements in Z1 and Z2 nanocomposites.

Sample	Atomic Percentages				
	% O	% Na	% Mg	% Al	% Si
Z1	59.3	8.1	8.6	3.4	20.6
Z2	55.2	11.5	6.0	4.9	22.4

**Table 2:** Experimental and kinetic model parameters for methylene blue dye adsorption onto Z1 and Z2 nanocomposites, including pseudo-first-order and pseudo-second-order model fitting.

Sample	Q_Exp_ (mg/g)	Pseudo-First-Order			Pseudo-Second-Order		
		K_1_ (1/min)	R^2^	Q_e_ (mg/g)	K_2_ (g/mg·min)	R^2^	Q_e_ (mg/g)
Z1	225.66	0.0262	0.9438	91.71	0.0007979	0.9999	220.75
Z2	285.28	0.0476	0.9894	62.59	0.0018770	0.9999	286.53

**Table 3:** Thermodynamic parameters for the adsorption of methylene blue dye onto Z1 and Z2 nanocomposites.

Sample	∆S^o^ (KJ/molK)	∆H^o^ (KJ/mol)	∆G^o^ (KJ/mol)			
			298	308	318	328
Z1	0.1308	−43.57	−82.55	−83.85	−85.16	−86.47
Z2	0.1215	−45.25	−81.46	−82.89	−83.89	−85.11

**Table 4:** Langmuir and Freundlich isotherm parameters for the adsorption of methylene blue dye onto Z1 and Z2 nanocomposites.

Sample	Langmuir			Freundlich			
	Q_max_ (mg/g)	R^2^	K_3_ (L/mg)	K_4_ (mg/g)(L/mg)^1/n^	Q_max_ (mg/g)	1/n	R^2^
Z1	230.95	0.9997	0.3077	91.31	258.53	0.1964	0.7219
Z2	290.69	0.9999	1.0886	144.36	345.05	0.1645	0.6641

**Table 5:** Maximum adsorption capacities (Q_max_) of various adsorbents for methylene blue dye.

Adsorbent	Q_max_ (mg/g)	Ref.
Activated coconut shells	30.30	[34]
Activated carbon/sodium lauryl sulfate composite	232.5	[35]
Cetyltrimethylammonium bromide/montmorillonite/polyaniline composite	61.30	[36]
ZnO/chitosan composite	97.93	[37]
Graphene oxide/ZnTiO_3_/TiO_2_ composite	78.00	[38]
ZSM-5 zeolite	105.82	[39]
NaX zeolite	24.39	[40]
Z1	230.95	This study
Z2	290.69	This study

**Table 6:** Experimental parameters for studying the effects of pH, time, temperature, and initial methylene blue dye concentration on adsorption.

Effect	V (L)	C_o_ (mg/L)	W (mg)	T (K)	t (min)	pH
pH	0.1	150	50	298	360	2.5–10.5
Time	0.1	150	50	298	10–100	10.5
Temperature	0.1	150	50	298–328	70 (Z1) 50 (Z2)	10.5
Concentration	0.1	50–300	50	298	70 (Z1) 50 (Z2)	10.5

## Data Availability

All data generated and analyzed during this study are included in this article.
